# Orbital Exostosis

**Published:** 1851-10

**Authors:** 


[*London Lancet.*

### ARTICLE XIX.

### *Orbital Exostosis.*

Mr. E. Canton, exhibited to the Medical Society of London, at the meeting in May, 1851, a specimen of orbital exostosis, which he had removed from a female, who applied to him at the Royal Westminster Ophthalmic Hospital. The patient is between twenty and thirty years of age, and in good health. She stated that seven months since, she noticed that her right eye began to protrude, and from that period to the present, the projection was steadily on the increase, at the same time that the organ was directed outwards. No pain was felt; vision was perfect; but the disfigurement was so detrimental to her as a servant, that she was anxious for its removal. On examination, it was found that besides the symptoms mentioned, the orbital ridge was increased in thickness, and a hard tumor, continuous with it, passed downwards, and deeply backwards into the orbit, so as to press upon the upper and back part of the eye, and cause a projection of the latter. Mr. Canton, having placed the patient under the influence of chloroform, made an incision from the external to the internal angular process of the frontal bone, in a semi-circular form, immediately below the eyebrow. The integuments, orbicularis muscle, and palpebral ligament being cut through, the dissection was continued into the orbit and around the tumor, so as to free the latter from neighboring and adherent soft parts. A small chisel was then used, in accessible situations to the bone of the tumor, which by degrees became detached from the orbital part, and was withdrawn from between the latter and the upper and lateral part of the eye. Sutures, plaster, and water dressing were applied, and the patient in a week recovered, not having had a bad symptom. The sight on the affected side, is nearly as perfect as on the sound one. The tumor is bony, about the size of a walnut, very heavy, and formed towards the periphery, of compact bone; whilst within the structure is of a close reticular character. The size of the base is equal to the surface of a shilling. The compact part of the tumor shows, under the microscope, some very large Haversian canals.—*London Lancet.*

